# Familiar Music Reduces Mind Wandering and Boosts Behavioral Performance During Lexical Semantic Processing †

**DOI:** 10.3390/brainsci15050482

**Published:** 2025-05-02

**Authors:** Gavin M. Bidelman, Shi Feng

**Affiliations:** 1 Department of Speech, Language and Hearing Sciences, Indiana University, Bloomington, IN 47408, USA; 2 Program in Neuroscience, Indiana University, Bloomington, IN 47405, USA; 3 Cognitive Science Program, Indiana University, Bloomington, IN 47405, USA; 4 Institute for Intelligent Systems, University of Memphis, Memphis, TN 38152, USA; 5 Center for Teaching and Learning, California State Polytechnic University, Humboldt, Arcata, CA 95521, USA

**Keywords:** music familiarity, mind wandering, lexical processing

## Abstract

Music has been shown to increase arousal and attention and even facilitate processing during non-musical tasks, including those related to speech and language functions. Mind wandering has been studied in many sustained attention tasks. Here, we investigated the intersection of these two phenomena: the role of mind wandering while listening to familiar/unfamiliar musical excerpts, and its effects on concurrent linguistic processing. We hypothesized that familiar music would be less distracting than unfamiliar music, causing less mind wandering, and consequently benefit concurrent speech perception. Participants (*N* = 96 young adults) performed a lexical-semantic congruity task where they judged the relatedness of visually presented word pairs while listening to non-vocal classical music (familiar or unfamiliar orchestral pieces), or a non-music environmental sound clip (control) played in the background. Mind wandering episodes were probed intermittently during the task by explicitly asking listeners if their mind was wandering in that moment. The primary outcome was accuracy and reactions times measured during the lexical-semantic judgment task across the three background music conditions (familiar, unfamiliar, and control). We found that listening to familiar music, relative to unfamiliar music or environmental noise, was associated with faster lexical-semantic decisions and a lower incidence of mind wandering. Mind wandering frequency was similar when performing the task when listening to familiar music and control environmental sounds. We infer that familiar music increases task enjoyment, reduces mind wandering, and promotes more rapid lexical access during concurrent lexical processing, by modulating task-related attentional resources. The implications of using music as an aid during academic study and cognitive tasks are discussed.

## 1. Introduction

Music listening is a ubiquitous part of everyday life. The pleasure of listening to music that we enjoy can improve emotional well-being [1], reduce stress and improve engagement in the workplace [2], increase emotional arousal [3], and may even increase some cognitive abilities including attention and executive functioning [4,5,6]. Moreover, music is often played during academic study or lectures in an attempt to influence student motivation and/or promote learning [7]. Conceivably, the positive (or negative) effects of music while performing other cognitive tasks might result from its ability to modulate task-oriented attention, emotional state, or arousal [7]. However, outside our own preliminary work [8], music seems to have been studied very little in psychological models of (in)attention.

Previous research suggests that our thoughts tend to ebb and flow between the mental states of orienting to both external and internal stimuli. One common internal thought state is mind wandering. Mind wandering describes the phenomenon when the processing of external stimuli are neglected in favor of processing internal task unrelated thoughts [9,10]. Using probe- and self-caught methods, studies show that mind wandering predicts errors on tasks that require sustained attention [11], word encoding [12], and even reading [13,14]. However, there has not been many studies on the role that mind wandering plays when one listens to background music while performing unrelated cognitive tasks (e.g., lexical decisions). Characterizing the tendency of mind wandering during music listening can inform current debates on whether music facilitates or disrupts language-related processing [15].

In the attentional resource model, operations that demand low attentional load provide more opportunities for mind wandering [9]. However, mind wandering resulting in attentional decoupling can also happen if a task is overly demanding, for example, when ones fails to construct a situation model for a difficult text when reading [14]. Another competing model is the attentional control model, which suggests that task disruptions due to mind wandering result from failures in executive maintenance [16,17]. This means that filtering task unrelated thoughts should be similar to filtering external distracting stimuli [18]. Unworth and McMillian (2014) [18] used a latent variable approach to measure participants on multiple constructs (e.g., working memory, fluid intelligence, and attentional control), and also sampled the participants’ attentional states with thought problems. Their results suggest that while mind wandering and external distractions partly covary with one another, they are distinct factors related to cognitive performance. Unworth and McMillian’s [18] research provides strong support for the attentional control hypothesis. Yet, an important question that arises is whether certain external stimuli (e.g., music) function as a distraction during task performance (prompting mind wandering), or alternatively, a mental facilitator (reducing mind wandering and thus increasing task performance).

An important aspect of music listening that needs to be taken into account is its familiarity [19]. The attentional-resource hypothesis [9] predicts that the more familiar the music, the more attentional resources are available for the mind to wander because familiar music is easier to process when performing a task. Increased mind wandering, in turn, leads to decreased task performance. Alternatively, by the attentional control hypothesis account [17], more familiar music should be less distracting to the task at hand, so individuals exert more attentional control, mind wander less, and show improved task performance [19].

Previous work on music and improving/disrupting task performance has shown conflicting results. Music listening disrupts performance if the music (e.g., vocal lyrics) shares semantic information with the primary task [20], even if the musical sound is irrelevant to the task. For example, listening to music with lyrics impairs reading comprehension more than listening to instrumental music (i.e., containing no lyrics) or silence [21]; this is true whether participants enjoy the lyrics or not. Similar findings have been reported by Brown & Bidelman (2022) [19] for open-set sentence recognition. While vocal music might have a predictable effect on concurrent language processing, whether instrumental music (without vocals) forces a similar determinant to behavior is an open question [19]. Instrumental music that is more familiar to a listener might still reduce competition on attentional resources between music listening and performance on goal-directed tasks.

Despite its potential effects on concurrent task performance, there is a surprising gap in the literature regarding the influence of background instrumental music and its familiarity on mind wandering, especially during lexical tasks. Here, we investigated the relationship between mind wandering and lexical performance while simultaneously listening to music. We also examined how familiarity with excerpts influences mind wandering and linguistic processing. We used a lexical-congruity task given the growing body of evidence suggesting a functional relationship between music and language processing [4,22]. However, unlike other elements of speech/language that share considerable overlap with the underlying brain resources supporting each domain (e.g., syntax [23,24,25,26] and acoustics [4]), the notion of semantic congruity is largely a phenomenon isolated to language (but see [27]). Indeed, the processing of music harmony shares cognitive resources related to syntactic analysis, whereas separate mechanisms are observed for semantic analysis [23]. Lexical-semantic congruity thus provides an interesting test of far transfer and competition of music for similar cognitive resources as language, given its uniqueness between domains. Anecdotally, music is often touted as a popular aid while studying [28]. However, experimental evidence is mixed on the impact of background music on non-musical task performance.

Our primary aims were to evaluate whether current music would facilitate or hinder concurrent lexical task performance depending on its familiarity to the listener (cf. [19,29]). We posited that if familiar music is less distractible, mind wandering frequency should increase, which in turn should predict faster lexical judgment response times and increased errors, per the attentional resource model [9]. An alternative hypothesis, per the attentional control model, argues there should be a decrease in mind wandering frequency, as less distractibility would lead to better attentional control and better performance in the concurrent lexical task.

## 2. Materials and Methods

### 2.1. Participants and Design

*N* = 96 undergraduate students from the University of Memphis participated in the study (mean age = 23.57 years, SD = 2.65). The stopping rule for determining sample size was based on the number of participants that completed the experiment during the 2-month period of data collection, with a goal of achieving at least 30 participants per condition to support the correlational analyses [30], and completing 32 full cycles of our counterbalance (see below). This sample is at least 4–6× the sample sizes used in similar studies regarding the effects of music on cognitive task performance [19,31], and accordingly was well powered. Subjects were excluded if they self-reported having hearing problems (e.g., hearing loss), a history of neuropsychological problems, or were not fluent in American English (all but two were native speakers). Participants were not informed about the purpose of the study ahead of time. Approximately 50% of the sample reported some musical training (mean years of musical training = 4.19 years, SD = 3.47). Participants gave written informed consent in compliance with a protocol approved by the Institutional Review Board of The University of Memphis.

The experiment used a within-subject design with three sound excerpt conditions played while the listeners performed a semantic congruity task. Words pairs were randomized within and between the participants. The three sound excerpt blocks were counterbalanced by a Latin square. Each subject received a different order of the three conditions (e.g., A-B-C; B-C-A; C-A-B). The full square was then repeated 32 times to satisfy the 96 person subject count. This effectively controlled order effects across the sample.

### 2.2. Measures

*Sound Excerpts.* Each participant received three musical familiarity conditions: control, familiar, and unfamiliar. The control condition consisted of a single sound clip, called the “environmental soundtrack,” which consisted of ambient nature sounds (e.g., birds chirping, trees rustling, and water flowing). This control condition was intended to mimic non-musical neutral background noise. Familiar and unfamiliar music consisted of a total of 12 classical musical excerpts by Bach, Mozart, and Beethoven; these were evenly distributed. Six were considered familiar and six were unfamiliar. The familiar pieces included Mozart’s Symphony # 40 in G minor (first movement), Beethoven’s Symphony # 5 in C minor, and Bach’s Cello Suite I in G major. Unfamiliar pieces included Mozart’s Serenade in B minor, Beethoven’s Violin Sonata # 10 in G major, and Bach’s Partita I for Solo Violin in B minor. In both the familiar and unfamiliar conditions, there were two musical excerpts per composer.

**Figure 1:**
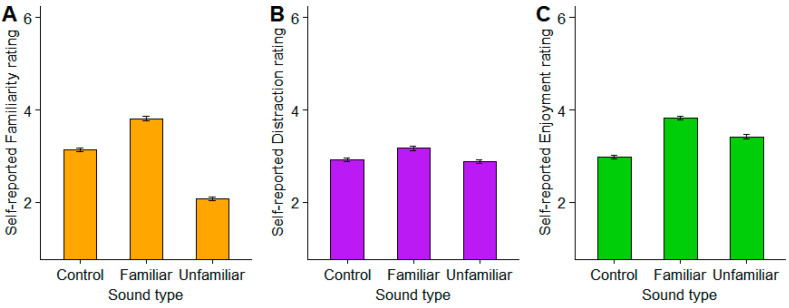
Participants’ self-reported (**A**) familiarity, (**B**) distraction, and (**C**) enjoyment ratings per background sound-type condition. Error bars = 95% CIs.

We initially selected these music excerpts *a priori* based on their popularity, and thus familiarity, reported in previous work [4,27]. However, we confirmed our experimenter selections were indeed more/less familiar to listeners during the actual experiment. The participants’ self-reported ratings of each condition, including familiarity, distractibility, and enjoyment, are shown in Figure 1. Importantly, the familiarity ratings confirmed a successful separation of familiar and unfamiliar music ratings, along with the neutral rating of the control condition. Familiarity (*F*(2, 17182) = 2700.2, *p* < 0.0001, $\eta_{p}^{2}=0.24$), distraction (*F*(2, 17182) = 76.88, *p* < 0.0001, $\eta_{p}^{2}=0.08$), and enjoyment (*F*(2, 17182) = 634.15, *p* < 0.0001, $\eta_{p}^{2}=0.07$) all varied across the three sound conditions.

Participants listened to one sound excerpt per condition, which was played while performing the semantic congruity task. The musical excerpt in the familiar and unfamiliar conditions featured one piece that was randomly selected from among the six possible choices in its category. While we selected the musical excerpts *a priori*, the self-reported familiarity ratings probed during the task confirmed that the subjects generally agreed with our experimenter-derived familiarity categories.

*Semantic Congruity Task*. The semantic congruity task was a word-pair semantic relatedness judgment task. There were a total of 180 word pairs from the appendix of Relander, Rama, and Kujala (2008) [32], English-translated version. For each of the three sound excerpt conditions, the participants were presented with 60 word pairs randomly selected from the 180 word pairs without duplication. In total, every participant completed the judgment of semantic relatedness of 180 word pairs (i.e., 60 per sound condition).

*Mind wandering sampling.* Mind wandering was occasionally assessed via the “probe caught” method [13,14] by inserting probes periodically into the task with an even distribution. There was a total of 15 probes per participant (5 per block), spaced every ~11 trials. The probe consisted of a screen shown between items of the lexical congruity task, with the question: “Are you mind wandering at this moment?” Participants clicked “yes” or “no” to signal whether they were mind wandering at that moment in the task. We did not ask participants for details on where their mind was wandering given the expected heterogeneity of responses, and the fact that we were interested in distractions away from the primary lexical task, not the nature of the mind wandering episodes, per se.

### 2.3. Procedure

The participants completed a demographic questionnaire regarding their education level, language background, and musical expertise (e.g., years of formal instrumental training). The participants were then prompted to complete the lexical-semantic task with experimental instructions presented on a PC monitor. The task required the participants to complete the series of word-relatedness judgments while listening to one of the three sound clips (unfamiliar music, familiar music, and environmental sound). The sounds were presented via circumaural headphones (Sennheiser HD 250) at a comfortable listening level (~70 dB SPL). Testing was performed in a quiet room to ensure the participants avoided any distractions during the experiment.

Before the task, participants read a definition of mind wandering taken from [9]: “Mind wandering is a term used to describe what occurs when your attention wanders from a task. Sometimes when your mind wanders, you begin thinking about personal events or concerns rather than your task. At other times, your mind can wander because you are bored or tired and you don’t really know what you’re thinking about; all you know is that you are no longer thinking about your task”. They were then told that they would periodically see messages asking them if they were mind wandering or not.

After each block, the participants were asked to rate the sound excerpt regarding familiarity (“How familiar are you with the clip that you’ve just heard?”), distractibility (“How distracting did you find the sound clip you’ve just heard?”), and enjoyment (“How much did you like listening to the sound clip you’ve just heard?”) using a 1–6 Likert Scale (see Figure 1). Response times on the word-pair judgments were also recorded, calculated as the time difference between the stimulus presentation (word pair) and the listeners’ behavioral response. The experimental task and data logging were coded in MATLAB (v2013; The MathWorks, Inc., Natick, MA, USA).

### 2.4. Statistical Analyses

Unless noted otherwise, we analyzed the dependent variables using generalized linear mixed-model ANOVAs in R (version 4.2.2) [33], the lme4 package [34]. Linear or logistic models were constructed based on whether the dependent variable was continuous (response times; *lmer*() function) or binary (mind wandering and accuracy of lexical-semantic judgment; *glmer*() function with binomial link function), respectively. In all models, the participants served as a random effect on intercept (96 levels). The sound conditions functioned as a three-level (familiar, unfamiliar, and control) categorical fixed effect. Multiple comparisons were corrected via Tukey–Kramer adjustments. For the main omnibus ANOVA results, the effect sizes are reported as partial eta squared ($\eta_{p}^{2})$ for lmer models and odds ratios (OR) for glmer models. The degrees of freedom (d.f.) were computed using Satterthwaite’s method. The significance level was set at α = 0.05 and two-tailed tests were used throughout.

## 3. Results

### 3.1. Lexical Semantic Judgments: Response Times and Accuracy

**Figure 2:**
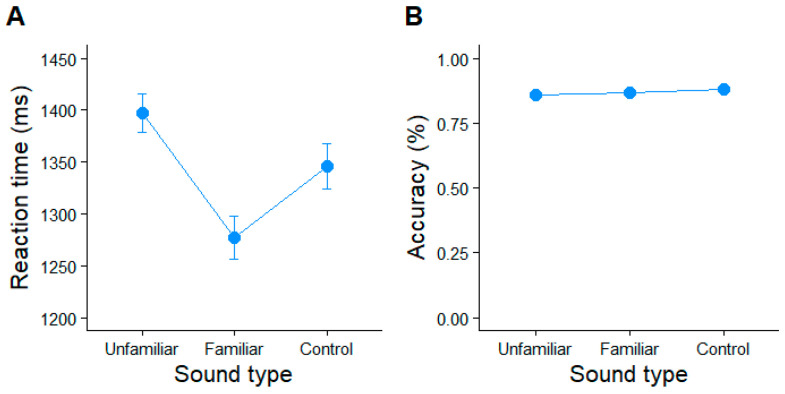
Mean (**A**) reaction time and (**B**) accuracy for the semantic judgments as a function of the familiarity of background music played currently during the linguistic task. Error bars = 95% CIs.

We first assessed if there were differences in the speed of current semantic judgments between the background music conditions, based on the listeners’ self-report stimulus familiarity. A mixed-effects ANOVA using the listeners’ self-reported familiarity to predict the semantic judgment response times was significant (*F*(2, 17121) = 62.56, *p* < 0.0001, $\eta_{p}^{2}=0.0073$; Figure 2A). Tukey-corrected post hoc contrasts revealed that the semantic judgment speeds were ~170 ms faster when listening to familiar vs. unfamiliar music. Similarly, the semantic judgements were 156 ms faster under familiar music than during the neutral (non-musical) control sound clips (*p* < 0.001). Response times did not differ between the unfamiliar music and control conditions (*p* = 0.70). These findings suggest participants were just as distracted while listening to the control sound as they were when performing the task during unfamiliar music.

The participants correctly judged 86% of the word pairs in semantic relatedness during the familiar music, 86% during the unfamiliar music, and 87% during the control sound (Figure 2B). A mixed-effects logistical ANOVA revealed that semantic judgment accuracy was invariant across the sound type conditions (*χ*2(2) = 1.06, *p* = 0.59). This suggests that the participants performed well (near ceiling), and that the accuracy in the lexical-relatedness judgments (unlike response speed) was not strongly modulated by the concurrent audio clip. These findings confirm our hypothesis that familiar music benefits concurrent speech perception primarily through faster (though equally accurate) lexical-semantic decisions.

### 3.2. Mind Wandering Frequency

**Figure 3:**
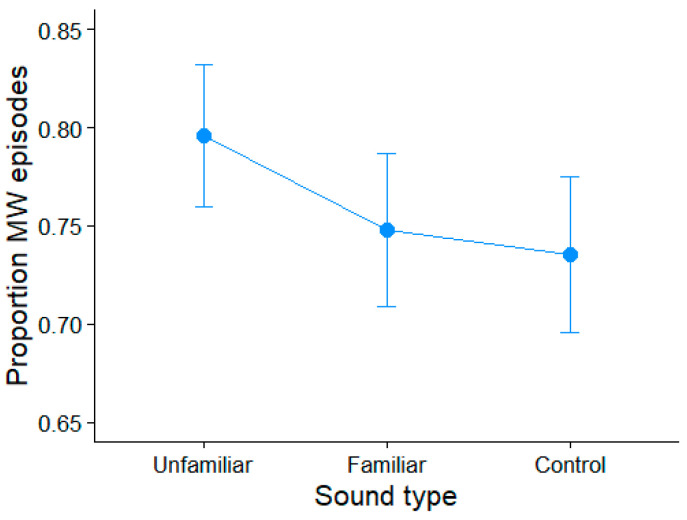
Proportion of mind wandering episodes per sound excerpt condition. Error bars = 95% CIs.

A mixed-effects logistics ANOVA for the presence/absence (coded as 1 and 0) of mind wandering revealed an effect of familiarity on mind wandering frequency (*χ*2(2) = 8.13, *p* = 0.0172). Participants were more likely to mind wander when performing the semantic relatedness task during the unfamiliar music than the control environmental sound (OR = 1.63; *p* = 0.0178) (Figure 3). Mind wandering did not increase under familiar music listening compared with the control condition (*p* = 0.84). However, mind wandering was more likely when listening to unfamiliar vs. familiar music (OR 1.48; *p* = 0.07). These findings generally support our hypothesis that familiar music causes less mind wandering than unfamiliar music and consequently benefits concurrent speech perception.

### 3.3. Musical Training, Familiarity, and Lexical-Semantic Response Times

**Figure 4:**
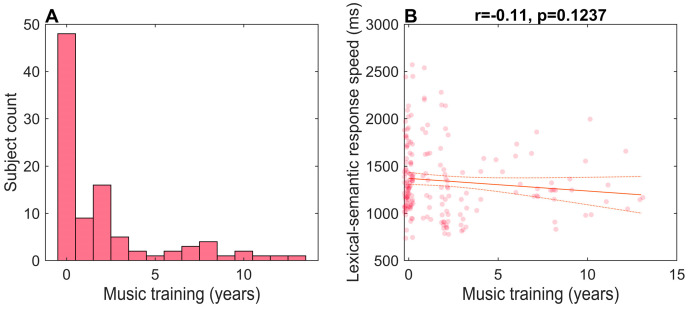
(Null) effects of musical training. (**A**) Histogram of the listeners’ self-reported music training. (**B**) Music training did not predict the response speeds in the lexical-semantic judgment task.

Musical training may augment language-related processing [4,35]. Given that 50% (48/96) of our cohort reported having at least 1 year of formal musical training (Figure 4A), we investigated whether musicianship might account for part of the variance in lexical response times. The correlations showed that formal musical training did not predict response speeds (*r* = −0.11, *p* = 0.12) in the lexical-semantic judgment task (Figure 4B). The lack of correlation is perhaps expected given that we did not explicitly recruit “musicians” and most of the sample had < 3 years of training (Figure 4A). Still, this rules out an explanation that prior musical training trivially drove the observed benefits of familiar music on lexical-semantic processing (i.e., Figure 2).

### 3.4. Musical Training, Familiarity Condition, and Mind Wandering

A mixed-effects logistical ANOVA on mind wandering (0 = absent, 1 = present) with musical training as a fixed effect (0 = absent, 1 = present), did not result in a model with improved fit compared to one with only the random effects (*χ*2(1) = 0.069, *p* = 0.79). The interaction between stimulus familiarity and musical training was also insignificant. These results indicate that participants experienced a similar frequency of mind wandering episodes regardless of their prior music training.

### 3.5. Familiarity, Distractibility, and Enjoyment

**Figure 5:**
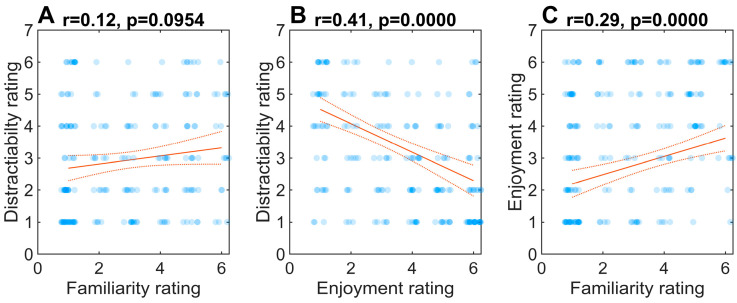
Correlations between the participants’ subjective ratings of (**A**) familiarity, (**B**) distractibility, and (**C**) enjoyment ratings of the background music conditions (control not included). Dotted lines = 95% CIs of the regression fit.

Lastly, we assessed the relationship between the ratings of familiarity, distractibility, and enjoyment of the music clips. Pearson’s correlations revealed no correspondence between music familiarity and distractibility (*r* = 0.12, *p* = 0.0954) (Figure 5A). Yet, how much the participants enjoyed the musical excerpts was negatively correlated with how distracting they perceived the musical excerpts to be (*r* = −0.41, *p* < 0.00001) (Figure 5B). The results also showed that, as with previous findings [19,36], the participants found the familiar music to be more enjoyable (*r* = 0.29, *p* < 0.00001) (Figure 5C). Collectively, these results indicate that the distractibility of a musical piece may not depend on how familiar that piece is to the listener. Instead, distractibility seems to be dependent on whether the listener enjoyed what they were hearing or not.

## 4. Discussion

We examined the effects of concurrent music, its familiarity, and mind wandering during cognitive processing required by language. We hypothesized that familiar music would be less distracting than unfamiliar music, causing less mind wandering, and consequently benefiting concurrent speech perception. Our results support these hypotheses. We found that listening to familiar music was less detrimental to lexical processing than listening to unfamiliar music, as evidenced by faster lexical-semantic decisions. The results also showed mind wandering was more likely when participants listened to unfamiliar music than either familiar music or background noise (i.e., neutral environmental sounds). Furthermore, prior musical training did not affect the speed of lexical decisions nor the frequency of mind wandering. Collectively, our results suggest that regardless of prior musical exposure (i) background audio that is unfamiliar (whether music or environmental noise) hinders language-related processing and increases mind wandering, and (ii) familiar music promotes more rapid lexical access and reduces mind wandering.

Our findings support previous work suggesting familiar music is less distracting than unfamiliar music [37,38] and can be beneficial to speech perception tasks [31]. Familiar background music might generate stronger expectancies that draw on fewer cognitive resources thereby enhancing speech recognition [31]. This provides stronger support for the attentional control model [17] rather than the attentional resources model [9]. Our findings agree with previous studies that suggest greater emotional arousal and pleasure in familiar music can alleviate task performance stress [2,3,36]. In our study, we found that listening to familiar music facilitated lexical-semantic decisions (i.e., faster decision speeds) compared with a neutral environmental soundtrack. Yet, response times when listening to unfamiliar music did not differ from listening to background noise.

Our findings diverge from some studies suggesting that familiar properties of music songs (especially vocals) negatively affect performance on a concurrent speech recognition task [19,29]. However, this and previous studies [29,31] used different types of music including pop songs, as well as different speech tasks (word identification and sentence recognition). Thus, it is possible the less complex demands of lexical-semantic decision (current study) compared with more complex sentence-level processing [19,39], might elicit different facilitatory or inhibitory effects of music. For example, while the neural tracking of speech is easier during familiar music, vocals in music have a larger impact on concurrent speech processing than instrumentals alone [29]. The effects of familiarity also depend on attention [39]. Our data here examining mind winding, agrees with these observations. Additionally, genre might play a role in modulating familiarity effects on speech perception. Indeed, pop songs (unlike the classic instrumental and environmental genres used here) evoke strong emotions and biographical memory [40], which might make them more distracting than our simple instrumental music, which we find can benefit performance (see also [19]). Indeed, distractibility was not related to familiarity with the purely instrumental music used here (Figure 5A). Future studies examining a variety of auditory-linguistic tasks and different genres of musical backdrops are needed to fully test the effects of music on concurrent speech processing.

While we replicated some previous findings that suggest unfamiliar music is more detrimental to task performance in simple speech perception tasks, they counter the notion that mind wandering reduces response times across the board [9]. In the current study, the participants’ listening to familiar music did not seem to shift their attentional resources allowing for their mind to wander, even though there was a reduction in response time; this is indicative of familiar music inducing task ease. Furthermore, the attentional resources model predicts that the participants with musical training should respond faster to stimuli when listening to familiar music and have more mind wandering episodes than the participants without musical training. Yet, we found musical training did not predict faster response times during familiar music conditions, nor did the mind of the “musicians” wander more. However, the null effects of musical training might be expected given that we did not explicitly recruit “musicians”. This aligns with other recent studies showing that the listener’s musicality (i.e., auditory perception skills), rather than their formal self-reported music training, governs how well they can juggle simultaneously presented speech and music sound streams [29,39].

In addition, correlational analyses suggested that familiarity might not have a clear-cut relationship with distractibility. This suggests that how enjoyable the music is to the listener might outweigh its familiarity in terms of driving task performance. Correlations did, however, show that familiarity was related to enjoyment. This replicates previous studies which have also found familiar music is more enjoyable [2]. Additionally, enjoyment was negatively correlated with distractibility. Thus, music familiarity and music preference may increase task ease by inducing pleasure, while alleviating mind wandering. The pleasure found in listening to music may be an added motivator for attention maintenance during tasks rather than an external distractor. Mind wandering is predominantly associated with boredom and negative moods [16,41,42]. Listening to preferred music may actually counteract boredom without compromising task performance.

Future research should assess the types of tasks that benefit from listening to familiar and/or preferred music. Music engagement has also been linked to creativity [43]. It would be worth investigating whether mind wandering occurs at a higher frequency while performing creative tasks during music listening relative to low-level, sustained attention tasks. We did not assess the participants’ musical genre preference, personality traits, or measure their baseline cognitive abilities. Participants were also monolingual speakers and from a uniform cultural background emersed largely in Western music. Such factors could be considered in future studies.

## 5. Conclusions

Music has been associated with better language processing [44,45], including improved lexical skills like reading [46,47]. Increasingly, listening to background music is being used as an academic study device to influence student motivation and/or promote learning [7,28]. Our study generally supports the notion that (familiar) music can facilitate certain aspects of language processing (lexical-semantic processing). Listening to music can be a creative endeavor in and of itself, and may spark ideas and encourage divergent thinking during unrelated tasks that would otherwise go unseen [48]. Future studies are needed to further investigate whether there are specific types of tasks or learning paradigms that can be aided (or hindered) by concurrent music listening and influenced by music training. Whether or not familiar music can benefit different learning environments, workplace productivity, clinical outcomes (e.g., using familiar music to aid focus in ADHD patients) or other cognitive tasks, remains to be studied.

## Data Availability

Data presented in this study are available on request from the corresponding author due to privacy reasons.
